# An exploration of healthcare use in older people waiting for and receiving Australian community‐based aged care services

**DOI:** 10.1111/ggi.14703

**Published:** 2023-10-20

**Authors:** Laura C. Edney, Hossein Haji Ali Afzali, Renuka Visvanathan, Barbara Toson, Jonathan Karnon

**Affiliations:** ^1^ Flinders Health and Medical Research Institute Flinders University Adelaide South Australia Australia; ^2^ Aged and Extended Care Services Queen Elizabeth Hospital and Basil Hetzel Institute, Central Adelaide Local Health Network Adelaide South Australia Australia; ^3^ Adelaide Geriatrics Training and Research with Aged Care (GTRAC) Centre Adelaide Medical School, University of Adelaide Adelaide South Australia Australia; ^4^ College of Medicine and Public Health Flinders University Adelaide South Australia Australia

**Keywords:** aged care, healthcare costs, home‐care package, older Australians, wait time

## Abstract

**Aim:**

Home care packages (HCPs) facilitate older individuals to remain at home, with longer HCP wait times associated with increased mortality risk. We analyze healthcare cost data pre‐ and post‐HCP access to inform hypotheses around the effects of healthcare use and mortality risk.

**Methods:**

Regression models were used to assess the impact of delayed HCP access on healthcare costs and to compare costs whilst waiting and in the 6‐ and 12 month periods post‐HCP access for 16 629 older adults.

**Results:**

Average wait time for a HCP was 89.7 days (SD = 125.6) during the study period. Wait‐time length had no impact on any healthcare cost category or time period. However, total per day healthcare costs were higher in the 6 and 12 months post‐receipt of a HCP (AU$61.5, AU$63, respectively) compared with those in the time waiting for a HCP (AU$48.1). Inpatient care accounted for a higher proportion of total healthcare costs post‐HCP (AU$45.1, AU$46.3, respectively) compared with in the wait time (AU$30.6), whilst spending on medical services and pharmaceuticals reduced slightly in the 6 month (AU$7.1, AU$6.3) and 12 month (AU$7.2, AU$6.3) post‐HCP periods compared with in the wait time (AU$7.9, AU$7.1).

**Conclusions:**

Increased spending post‐HCP on inpatient care or non‐health support afforded by HCPs may offer protective effects for mortality and risk of admission to aged care. Further research should explore the association between delayed access to inpatient care for geriatric syndromes and mortality to inform recommendations on extensions to residential care outreach services into the community to improve the timely identification of the need for inpatient care. **Geriatr Gerontol Int 2023; 23: 899–905**.

## Graphical abstract

We find no impact of wait time on healthcare costs, but inpatient geriatric care was higher post‐HCP while primary and other forms of secondary care declined.

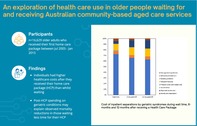

## Introduction

The number of people aged 65 years and over is increasing, with older people representing over 15% of the Australian population.^1^ Many require assistance to meet their needs, such as community‐based services or admission into an aged care facility,^2^ with community‐based services often preferred.^3^ To support this, the Australian Government subsidizes a range of community‐based aged care services such as home care packages (HCPs) to assist people at home. These HCPs provide personal care and domestic assistance needs^4^ through the provision of social (e.g., transport and social activities), practical (e.g., household chores and maintenance, garden maintenance, home modifications and other independence aids) and health‐related (e.g., nursing, allied health, transport to health appointments) support.^5^ Over half of HCP funds are allocated to these care services, with significant proportions directed toward administration.^6^ To determine HCP eligibility, the Aged Care Assessment Team (ACAT) consider medical, physical and psychosocial needs and the presence of existing support systems.^7^ During the period of our study, HCPs resided with care providers, and eligible individuals would wait for their package to be assigned by clinicians employed by the care provider. As of 30 September 2021, there were 74 143 individuals waiting for their approved HCP level, with estimated wait times of between 3 and 9 months.^8^ Among those waiting, approximately 20% had accepted a lower‐level HCP, and nearly all (73 279) had been offered the opportunity to connect to some form of Commonwealth‐subsidized home‐care support. Given the role of HCPs in preventing poor health through addressing health‐ and social‐care needs, delayed access may adversely affect health outcomes and increase the risk of declining function, hospital admissions and mortality.

An Australian study reported that longer wait times for a HCP were associated with a higher risk of mortality and that waiting for more than 6 months was associated with a 10% higher risk of moving to residential aged care services 2 years after receiving the HCP.^9^ There is no empirical evidence on the impact of wait time on the use of healthcare resources and costs to the healthcare system. Healthcare costs for older Australians have been projected to increase to $180 billion in 2035,^4^ so understanding the costs to meet the long‐term health needs of this population to support sustainable policy is essential.

This study aims to generate new evidence on the impacts of delayed access to a HCP. We aim to describe healthcare utilization whilst waiting and in the 12 months post‐HCP and to assess whether those waiting longer for a HCP incur additional healthcare costs whilst waiting and post‐HCP. Given the previously identified mortality effects of longer wait times,^9^ we hypothesize that longer HCP wait times will be associated with higher healthcare costs.

## Methods

### Study cohort

This is a retrospective cohort study using deidentified individual data from the Registry of Senior Australians Historical Cohort (ROSA).^10^ The study cohort includes South Australians (65+ years) who had approval for and had received their first HCP between July 2005 and January 2015. Individuals who did not receive a HCP within these dates were excluded, including those who had no service, those still waiting for their HCP, and those who entered permanent residential aged care prior to receiving a HCP. Before 2013, home‐based care services were categorized into different levels, including Community Aged Care Packages (CACPs), Extended Age Care at Home (EACH) and Extended Aged Care at Home Dementia (EACH‐D) packages. HCPs replaced these categorizations in 2013, but for the purposes of this study, home‐based care services will collectively be referred to as HCPs.

### Variables

#### Exposures

Wait time was the exposure of interest and was defined as the time between the date of ACAT assessment approval and the date of first access to a HCP, at any level.

Covariates included age, gender, marital status, country of birth, remoteness, living arrangements, HCP level, carer availability, concession status, ACAT location, and a range of health status covariates to capture health need including health conditions, activity limitations, frailty and the presence of polypharmacy (≥10 prescription medications at time of approval) based on ACAT assessment and medication use and healthcare utilization prior to ACAT assessment.

#### Outcomes

Healthcare utilization and costs, calculated as costs incurred (i) whilst individuals were waiting for their HCP, (ii) 6 months post‐HCP access, and (iii) 1 year post‐HCP access, were captured from administrative data sources in ROSA.

##### Medicare benefits schedule and pharmaceutical benefits scheme

Spending on subsidized medical services listed on the Medicare benefits schedule (MBS) was captured through government benefit paid to healthcare providers. Spending on pharmaceuticals was obtained from the government benefit paid in each given year toward subsidized pharmaceuticals listed on the pharmaceutical benefits scheme (PBS).

##### Public hospital inpatient separations

Australian refined diagnosis‐related group (AR‐DRG) codes were used to assign hospital separation 2018–19 price weights. Inpatient separations were disaggregated by geriatric syndromes (dementia and delirium, mobility problems, falls and fractures, pressure ulcers and weight loss, incontinence, dependence and care, anxiety and depression) defined by International Classification of Diseases 10th Revision (ICD‐10) codes^11^ and external cause of injury.

##### Public hospital Emergency Department presentations

Metropolitan Emergency Department (ED) presentations were presented by Urgency Related Groups (URGs). Price weights associated with each URG and adjustment factors in 2018–19 were multiplied by the National Efficient Price in the relevant years not weighted for Indigenous status.

### Data analysis

Data were checked for missing values and outliers, but no extreme values were excluded. Separate generalized linear models (GLMs) with a log link function and gamma distribution^12^ were used to account for the potential non‐linear impact of covariates and right‐skewed cost data. Models were estimated to test the hypothesis rather than to identify the best model to explain variability in healthcare costs. The criterion for statistical significance was 5%, and all analyses were performed with StataSE 16.0 (StataCorp,College Station, TX, USA).

Additional planned sensitivity analyses included (1) model re‐specification using a two‐part model (a probit model to predict the probability of incurring costs, followed by a GLM model with a log link function and gamma distribution to model spending, conditional on any spending); (2) costs incurred during wait time as a covariate in models predicting post‐HCP costs; (3) stratified analyses for (i) individuals who remained alive throughout the study, (ii) individuals with ACAT assessment outside of hospital, and (iii) individuals who received lower HCP levels indicating less need; and (4) quantile regression analyses to estimate wait‐time effects across quantiles of healthcare spending.

## Results

The final study sample with complete case data consisted of 16 629 older people (mean age 82.9 years; SD = 6.8 years; 67.6% female). The average time between ACAT assessment and receiving a HCP at any level was 89.73 days (SD = 125.58 days), with 39.61% receiving their HCP within 30 days and 11.17% within 7 days.

Baseline characteristics of the sample by mean wait time for an HCP and healthcare costs are reported in Table 1. Greater average wait times for a HCP were experienced by males, those with a carer, and those living with family, with shorter wait times for females, those without a carer, and those living alone or with others excluding family.

**Table 1:** Descriptive characteristics of the study cohort by length of time waiting for a HCP and healthcare costs whilst waiting and in the 6 and 12 months post‐HCP access

	Total sample	Wait time (days)	Mean total healthcare costs (AU$) per day		
			Wait time	6 months post‐HCP period	12 months post‐HCP period
	*N* (%)	*M* (SD)	*M* (SD)	*M* (SD)	*M* (SD)
Aggregate	16 614 (100)				
Gender					
Male	5379 (32.4)	92.8 (134.8)	57.5 (224.1)	77.0 (230.8)	80.3 (195.9)
Female	11 235 (67.6)	88.3 (121.0)	43.6 (231.6)	54.1 (128.2)	54.7 (114.6)
HCP service received					
CACP	10 808 (65.0)	80.2 (94.5)	46.4 (245.1)	60.0 (184.4)	61.2 (149.2)
EACH	589 (3.5)	124.2 (154.0)	56.4 (158.3)	78.9 (143.9)	91.2 (247.7)
EACHD	571 (3.4)	103.2 (128.2)	35.5 (98.9)	61.4 (131.6)	61.4 (133.0)
Level 1	148 (0.9)	85.9 (163.4)	34.7 (107.6)	47.8 (100.8)	50.9 (96.8)
Level 2	3892 (23.4)	101.0 (168.0)	53.1 (223.0)	62.3 (135.8)	63.2 (123.9)
Level 3	199 (1.2)	82.3 (112.9)	53.3 (114.2)	73.6 (131.2)	83.4 (140.5)
Level 4	422 (2.5)	167.6 (225.1)	51.6 (120.2)	72.2 (133.6)	66.4 (110.5)
Carer					
Has carer	13 940 (83.9)	92.0 (129.6)	47.2 (224.7)	61.4 (173.6)	62.9 (147.7)
Has no carer	2561 (15.4)	77.2 (100.4)	51.6 (256.2)	62.8 (143.1)	64.2 (143.5)
Living arrangements					
NA	78 (0.5)	75.1 (91.1)	82.1 (229.3)	79.8 (120.0)	76.6 (107.7)
Lives alone	9053 (54.5)	81.6 (111.9)	48.7 (272.0)	56.5 (123.1)	58.7 (126.4)
Lives with family	7193 (43.3)	100.0 (140.8)	47.0 (164.6)	66.2 (153.3)	67.8 (153.2)
Lives with others	280 (1.7)	89.8 (113.4)	47.0 (128.5)	105.0 (770.7)	78.5 (390.4)
Movement activity limitations					
Has limitation	3134 (18.9)	101.4 (142.9)	71.9 (374.8)	80.8 (276.5)	79.5 (202.0)
None	13 488 (81.1)	87.0 (121.1)	42.5 (178.8)	57.2 (131.4)	59.2 (130.2)
NA	7 (0.0)	73.6 (65.2)	42.9 (78.1)	38.4 (51.7)	22.7 (25.2)
Polypharmacy (10+)	6984 (42.0)	89.6 (128.3)	62.9 (268.8)	75.2 (152.9)	75.8 (135.4)
No	9645 (58.0)	89.8 (123.6)	37.3 (194.9)	51.8 (178.8)	53.9 (153.6)
Number of health conditions					
0	553 (3.3)	98.8 (128.5)	26.1 (84.2)	39.7 (90.8)	40.4 (80.7)
1	2028 (12.1)	89.3 (119.1)	36.9 (97.8)	53.2 (148.7)	58.2 (181.5)
2	3777 (22.7)	86.7 (123.3)	45.1 (183.7)	61.6 (240.6)	64.6 (186.2)
3	4352 (26.1)	90.8 (124.6)	48.3 (242.0)	61.0 (136.7)	61.0 (120.8)
4	3238 (19.5)	89.7 (130.6)	54.3 (312.8)	65.3 (164.4)	65.1 (132.0)
5	1723 (10.4)	89.7 (127.5)	55.1 (168.2)	66.2 (114.0)	68.2 (115.1)
6	729 (4.4)	89.2 (124.4)	62.3 (369.6)	71.6 (120.7)	71.4 (115.2)
7	203 (1.2)	109.7 (153.6)	52.0 (88.1)	76.2 (127.3)	74.6 (115.7)
8	37 (0.2)	71.7 (83.4)	89.2 (149.1)	75.1 (113.0)	82.8 (137.2)
9	6 (0.0)	30.3 (18.0)	19.0 (22.7)	77.4 (84.1)	46.2 (44.8)
Frailty status					
Frail (>0.21)	6942 (41.7)	89.9 (121.7)	38.1 (124.3)	53.3 (200.1)	54.1 (136.5)
Not frail/robust	9704 (58.3)	89.6 (128.3)	55.3 (281.0)	67.4 (141.9)	69.4 (153.0)

Abbreviations: AU$, Australian dollars; CACP, community aged care package; EACH, extended aged care at home; EACHD, extended aged care at home‐dementia; HCP, healthcare package.

In general, per day total healthcare costs were highest in the 12 month post‐HCP entry period and lowest during wait time, regardless of duration waiting. Higher healthcare costs were observed across all time periods for males and for those with health conditions compared with those without. Costs were lower across all three time periods for those with no reported health conditions and for those with the presence of some health conditions, including dementia, arthritis, hearing and eye conditions. Healthcare costs were similar across all three time periods for those reporting falls compared with those without, and whilst we saw higher costs in the two post‐HCP periods for those that live with family compared with those that live alone, we saw similar costs between groups during the wait time for their HCP.

### Healthcare costs

The mean total per day healthcare costs during wait time was AU$48.1, of which the majority (63.6%) was incurred through inpatient separations, with smaller costs incurred for MBS services (AU$7.9, 16.4%), PBS scripts (AU$7.1, 14.8%) and ED presentations (AU$2.5, 5.2%) (see Table 2).

**Table 2:** Healthcare costs (AU$) per day whilst waiting for a HCP and for the 6‐ and 12 month post‐HCP access periods

	Wait time		6 month post‐HCP period		12 month post‐HCP period	
	M	SD	M	SD	M	SD
Hospital costs						
Public hospital inpatient costs	30.6	216.2	45.1	164.2	46.3	141.9
Inpatient costs, including MBS in hospital costs	32.4	216.7	46.8	164.8	48.1	142.5
Inpatient cost categories						
Geriatric syndrome	8.4	109.9	15.4	134.8	17.2	110.7
Not a geriatric syndrome	22.2	185.3	29.7	90.3	29.2	85.1
Dementia and delirium	1.6	62.6	2.2	18.8	2.4	17.1
Mobility problems	0.6	64.6	0.1	3.2	0.1	2.5
Falls and fractures	3.3	40.9	6.2	33.7	6.4	28.8
Pressure ulcers and weight loss	0.1	5.1	0.1	3.0	0.1	3.1
Incontinence	0.1	3.2	0.1	0.9	0.1	0.5
Dependence and care	1.5	38.1	5.4	125.3	6.9	101.6
Anxiety and depression	1.3	28.5	1.4	22.6	1.2	17.4
ED costs	2.5	20.0	3.1	8.7	3.2	8.4
Inpatient and ED costs	33.1	225.7	48.1	167.1	49.5	144.9
Medicare costs (excluding in hospital costs)						
MBS	7.9	15.6	7.1	9.5	7.2	9.0
PBS	7.1	23.4	6.3	10.7	6.3	10.5
MBS and PBS	15.0	29.1	13.4	15.6	13.5	15.1
Total costs (inpatient, ED, MBS, PBS)	48.1	229.2	61.5	168.8	63.0	146.6

Abbreviations: ED, emergency department; HCP, homecare package; MBS, Medicare benefits schedule; PBS, pharmaceutical benefits scheme.

Total per day healthcare costs were higher in the 6 (AU$61.5) and 12 (AU$63) months following HCP access. Patterns of high inpatient costs and low ED presentation costs were observed in both post‐HCP time periods. However, the cost of inpatient separations as a proportion of total healthcare costs was higher in the post‐HCP periods (6 months: 73.3%; 12 months: 73.5%) compared with in the wait time (63.6%), whilst MBS (6 months: 11.5%; 12 months: 11.4%) and PBS (6 months: 10.2%; 12 months: 10%) were lower in the post‐HCP periods compared with in the wait time (MBS: 16.4%; PBS: 14.8%).

### Inpatient separations

**Figure 1:**
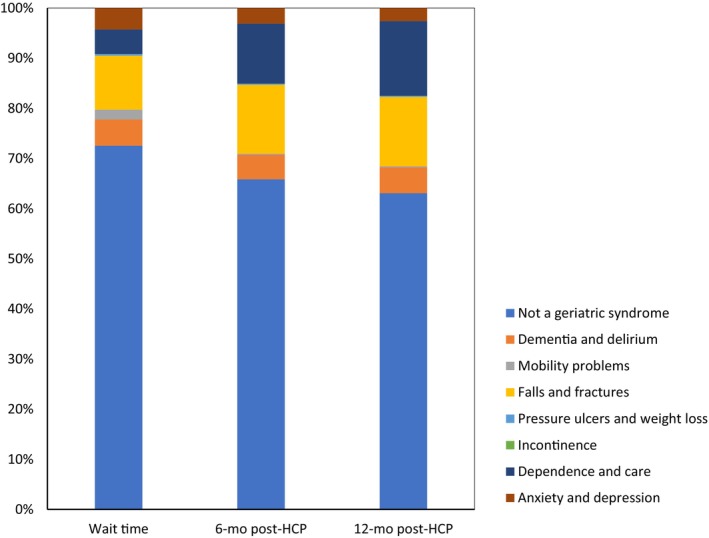
Cost of inpatient separations by geriatric syndromes during wait time and in the 6 and 12 months after receiving a healthcare package (HCP).

The majority of costs incurred through inpatient separations were not associated with a geriatric condition (see Table 2 and Fig. 1). However, costs associated with separations recorded to a geriatric syndrome were higher in the two post‐HCP time periods compared with during the wait time, largely driven by higher inpatient costs due to falls and fractures and dependence and care, which includes the need for assistance owing to reduced mobility, the need for assistance with personal care, and problems related to care provision, including the unavailability of alternative medical services and awaiting admission to an alternative facility. There were slightly reduced costs observed for inpatient costs due to mobility problems in the 6 and 12 month post‐HCP periods compared with during the wait time.

### MBS items

Approximately three‐quarters of MBS spending was on out‐of‐hospital spending across all time periods (wait time = 77.2%; 6 months = 76.1%; 12 months = 75%). Unreferred GP services accounted for 35% of the total MBS service costs (wait time = 35.4%; 6 months = 35.2%; 12 months = 36.1%) and for a slightly higher proportion of total healthcare costs during wait time (5.8%) compared with the 6‐ (4.1%) and 12 month (4.1%) post‐HCP periods.

### Impact of length of delay waiting for a HCP on healthcare costs

Coefficients on wait time, representing the impact of number of days waiting for a HCP on each category of healthcare cost controlling for covariates, are reported in Table 3. Wait time had no meaningful impact on any measure of healthcare costs incurred during wait time or in either of the post‐HCP periods.

**Table 3:** Impact of length of wait time on healthcare costs (AU$/day) across each outcome model

	Wait time		6 month post‐HCP period		12 month post‐HCP period	
	*β*	95% CIs	*β*	95% CIs	*β*	95% CIs
Hospital						
Inpatient	0.0001	−00004, 0.0007	−0.0003	−0.0007, 0.0000	−0.0002	−0.0005, 0.0001
Inpatient and MBS in hospital	−0.0000	−0.0005, 0.0004	−0.0003	−0.0007, 0.0000	−0.0002	−0.0005, 0.0001
Inpatient and ED	0.0000	−0.0005, 0.0005	−0.0003	−0.0006, 0.0001	−0.0002	−0.0005, 0.0001
Medicare						
MBS^†^	−0.0007	−0.0008, −0.0005	−0.0001	−0.0002, 0.0001	−0.0000	−0.0001, 0.0001
PBS	−0.0004	−0.0006, −0.0001	0.0000	−0.0001, 0.0002	0.0000	−0.0002, 0.0002
MBS and PBS	−0.0006	−0.0007, −0.0004	−0.0000	−0.0001, 0.0001	−0.0000	−0.0001, 0.0001
Total costs (hospital and Medicare)	−0.0004	−0.0007, −0.0001	−0.0003	−0.0005, −0.0000	−0.0002	−0.0004, 0.0000

All models adjusted for: gender, HCP service received, carer availability, living arrangements, movement activity limitations, polypharmacy (10+), number of health conditions, frailty status, concession card type, country of birth, remoteness classification, approvals for respite care, permanent residential care and transition care, and the index of relative socioeconomic disadvantage. CI, confidence interval; ED, emergency department; HCP, healthcare package; MBS, Medicare benefits schedule; PBS, pharmaceutical benefits scheme.

† Excluding MBS items incurred in‐hospital.

### Sensitivity analyses

Sensitivity analyses confirmed the main findings when stratified by mortality, ACAT location, HCP level, controlling for wait‐time costs, or respecified in two‐part models. In quantile regressions, wait time had small significant coefficients (range: *β* = −0.0168, *β* = 0.0357) across the full distribution of healthcare costs, suggesting a minimal differential impact of wait time for either high‐ or low‐healthcare users (see Tables A1 and A2, Supporting Information).

## Discussion

Many older Australians experience delays in receiving a HCP, with only 14% of individuals receiving a HCP within a month.^13^ This study reported no impact of length of time waiting for a HCP on healthcare costs, but that inpatient costs increased post‐HCP access regardless of wait‐time length, driven by increased spending in geriatric syndromes of falls and fractures and dependence in care, and, to a lesser extent, in dementia and delirium. Spending on medical services and pharmaceuticals outside of hospitals decreased post‐HCP access. Inpatient spending on other geriatric syndromes, non‐geriatric syndromes, and ED presentations did not increase post‐HCP.

Increased spending on dependence and care, which includes separations where alternative care facilities are unavailable or where there is a need for assistance with personal care, may be warranted and due to deconditioning prior to ACAT assessment and whilst waiting for a HCP. This deconditioning may require escalated care and be triggered by increased identification of health needs by HCP service providers. During the study period, ACATs were linked to publicly funded geriatric medicine services, with geriatricians involved in many ACAT teams, both conducting assessments and participating in discussions about assessed individuals. This relationship might have been a contributor to increased post‐package identification as healthcare needs were identified. The lack of increase in primary care post‐HCP suggests that general practitioners (GPs) may be underutilized; this is supported by prior evidence for this cohort, with only 5% of general practice services involving comprehensive assessment.^14^ GPs can play an important role in identifying and managing geriatric conditions.^15^ Increased spending following receipt of a HCP, predominantly through increased hospitalization costs rather than through general practice use, raises the possibility that improved integration between general practice and aged care services might reduce the over‐reliance on reactive, hospital‐based care. Reorientating healthcare for HCP recipients, and prioritizing healthcare for older individuals, particularly those approved but waiting for their HCP, toward general practice and primary care would align with the World Health Organization's guidelines for Integrated Care for Older People.^16^ The increased spending on falls and fractures for subcategories of osteoporosis, tendency to fall and senility may similarly be due to increased identification of need and access to care facilitated by HCP service providers. Increased spending for subcategories of falls, fractures, dislocations, sprains and strains may also be due to increased mobility facilitated by the HCP through practical (e.g., mobility aids) and social (e.g., increased social outings) support. ED presentations and primary and secondary health services did not increase post‐HCP, suggesting that increased inpatient care post‐HCP may not be facilitated via these routes; referrals for inpatient care may have been through outpatient departments, private medical practices or community health services.

The delayed effect of longer wait times for a HCP on mortality and risk of admission to permanent aged care identified previously^9^ is consistent with people who wait longer for a HCP experiencing a delay in healthcare. However, we may not have observed a meaningful effect of wait time on healthcare costs owing to unobserved confounding. During the study period, HCPs were allocated to providers along with a list of approved individuals. There may be an unobserved clinician effect used to prioritize individuals that not captured in our control variables. A National Priority System was introduced in 2017 for all approved individuals, with HCPs now assigned to the individual rather than to the provider; therefore, similar analyses with more recent cohorts may not be confounded by clinician provider effects. Although we report no impact of wait time for a HCP on healthcare costs, it could be that any delay in receiving geriatric care impacts later mortality and admission to aged care, as supported by evidence that suggests that geriatric syndromes are associated with hospitalizations^17^, ^18^, ^19^ and increased risk of mortality.^20^, ^21^ Alternatively, the delayed mortality and aged care admission effects from extended wait times for a HCP may be due to health impacts delivered by the practical and social aspects of HCPs, rather than to increased healthcare use associated with HCPs. Evidence regarding the impact of social support on mortality is mixed,^22^ although social support may moderate the relationship between physical multimorbidity and mortality,^23^ and poor social health may have a direct impact on cardiovascular mortality.^24^

Future research should seek to evaluate the impact that categories of healthcare spending and of care provided through the HCP have on morbidity, mortality and transition to permanent aged care. Confirmation of the protective effect of earlier access to inpatient geriatric care could inform the extension of current residential care outreach services (e.g., Ref. 25, 26, 27) into the community (e.g., Ref. 28) or primary care (e.g., Ref. 14) for earlier identification of need. Timely access to services, including HCPs and healthcare, are essential. Since this study period there has been a Royal Commission into Aged Care Quality and Safety. Based on their recommendations, the Australian government have committed to clearing current wait lists and reducing future wait times to less than 1 month through an additional AU$6.48 billion to support 275 600 HCPs. We further recommend improved access to primary care for older individuals and, in particular, that entering a wait list for an approved HCP should trigger primary care prioritization through the use of telehealth or home‐care visits to prevent deconditioning and to improve the timely identification of need for inpatient geriatric services.

This study is the first to quantify the impact of wait time for a HCP on healthcare costs, but several limitations must be considered. The current study analyzes data prior to the implementation of the National Prioritisation List and prior to the Royal Commission into Aged Care Quality and Safety. Analyses were limited to change in costs over the 6 and 12 month post‐HCP periods for those who received a HCP. We recognize that spending may be immediately higher in the days and weeks after receiving the HCP; however, any such immediate increases that are not sustained are of less policy relevance. Our analyses also excluded individuals who were still waiting for their HCP and any private healthcare use, including any use of private hospitals, private allied health or privately funded aged care services.

## Conclusions

The study found no impact of wait time on healthcare costs, but inpatient geriatric care was higher post‐HCP while primary and other forms of secondary care declined. Prior evidence reported increased mortality risk and admission to permanent residential aged care from longer wait times,^9^ suggesting that post‐HCP spending on geriatric conditions or non‐health support afforded by HCPs may be offering protective effects when received earlier by those waiting less time for their HCP. Further research should explore the association between delayed access to inpatient care for geriatric syndromes and mortality in order to inform recommendations on potential extensions to residential care outreach services into the community to improve timely identification of the need for inpatient care.

## Disclosure statement

None of the authors have any conflicts of interest to declare.

## Ethics statement

This study obtained ethics approvals from the University of South Australia Human Research Ethics Committee (Ref: 200489), the Australian Institute of Health and Welfare Ethics Committee (Ref: EO2022/4/1376) and the South Australian Department for Health and Wellbeing Human Research Ethics Committee (Ref: HREC/18/SAH/90) for the inclusion of South Australia datasets.

## Supporting information


**Table A1.** Impact of length of wait time on healthcare costs across different model specifications (AU$/day).
**Table A2.** Impact of length of wait time on healthcare costs across quantiles of spending (AU$/day).

## Data Availability

No data are available. These data were made available to the researchers under ethical, governance and confidentiality agreements that do not allow public sharing. The data that support the findings of this study are available from the Registry of Senior Australians (ROSA). Restrictions apply to the availability of these data, with approvals required from data custodians owing to privacy and ethical considerations.
