# Supplementary material for: An exploration of healthcare use in older people waiting for and receiving Australian community‐based aged care services
Source: Geriatr Gerontol Int. 2023 Oct 20;23(12):899–905. doi: 10.1111/ggi.14703 (PMC11503569; doi:10.1111/ggi.14703)
Supplement: Supplementary file 1 — Table A1. Impact of length of wait time on healthcare costs across different model specifications (AU$/day). Table A2. Impact of length of wait time on healthcare costs across quantiles of spending (AU$/day). [file GGI-23-899-s001.docx]

**Supporting Information: Sensitivity Analyses**

**Table A1.** Impact of length of wait time on healthcare costs across different model specifications (AU$/day).

|  | Wait time | | 6-mo post HCP | | 12-mo post HCP | |
| --- | --- | --- | --- | --- | --- | --- |
|  | **β** | **SE** | **β** | **SE** | **β** | **SE** |
| Controlling for healthcare costs during wait time |  |  |  |  |  |  |
| MBS and PBS | - | - | 0.0000 | 0.0001 | 0.0000 | 0.0000 |
| Total costs (inpatient, ED, MBS, PBS) | - | - | -0.0003 | 0.0001 | -0.0002 | 0.0001 |
| Stratified analyses. Analyses restricted to individuals who: |  |  |  |  |  |  |
| 1. Remained alive throughout the study period |  |  |  |  |  |  |
| MBS and PBS | -0.0006 | 0.0001 | -0.0000 | 0.0001 | 0.0000 | 0.0001 |
| Total costs (inpatient, ED, MBS, PBS) | -0.0003 | 0.0002 | -0.0002 | 0.0001 | -0.0001 | 0.0001 |
| 1. Had their ACAT conducted outside of hospital |  |  |  |  |  |  |
| MBS and PBS | -0.0006 | 0.0001 | -0.0000 | 0.0001 | -0.0000 | 0.0001 |
| Total costs (inpatient, ED, MBS, PBS) | 0.0001 | 0.0002 | -0.0002 | 0.0001 | -0.0001 | 0.0001 |
| 1. Received CACP HCP level |  |  |  |  |  |  |
| MBS and PBS | -0.0010 | 0.0001 | -0.0001 | 0.0001 | -0.0000 | 0.0001 |
| Total costs (inpatient, ED, MBS, PBS) | -0.0004 | 0.0003 | -0.0004 | 0.0002 | -0.0003 | 0.0002 |
| 1. Received Level 2 HCP level |  |  |  |  |  |  |
| MBS and PBS | -0.0004 | 0.0002 | -0.0001 | 0.0001 | -0.0001 | 0.0001 |
| Total costs (inpatient, ED, MBS, PBS) | -0.0002 | 0.0002 | 0.0001 | 0.0003 | 0.0003 | 0.0002 |

*ACAT, Aged Care Assessment Team; CACP, Community Aged Care Package; ED, Emergency Department; HCP, Home Care Package; MBS, Medicare Benefits Schedule; PBS, Pharmaceutical Benefits Scheme*

**Table A2.** Impact of length of wait time on healthcare costs across quantiles of spending (AU$/day).

|  | Quantiles |  |  |  |  |  |  |  |  |  |
| --- | --- | --- | --- | --- | --- | --- | --- | --- | --- | --- |
| DV: costs | **0.1** |  | **0.25** |  | **0.5** |  | **0.75** |  | **0.9** |  |
|  | **β** | **SE** | **β** | **SE** | **β** | **SE** | **β** | **SE** | **β** | **SE** |
| Wait time |  |  |  |  |  |  |  |  |  |  |
| MBS and PBS | 0.0087 | 0.0006* | 0.0070 | 0.0005* | 0.0025 | 0.0006* | -0.0006 | 0.0012* | -0.0168 | 0.0031* |
| Total costs | 0.0130 | 0.0007* | 0.0150 | 0.0007* | 0.0189 | 0.0012* | 0.0357 | 0.0047* | 0.0151 | 0.0212 |
| 6mo post HCP |  |  |  |  |  |  |  |  |  |  |
| MBS and PBS | 0.0001 | 0.0004 | 0.0001 | 0.0004 | -0.0001 | 0.0005 | -0.0003 | 0.0008 | 0.0002 | 0.0020 |
| Total costs | -0.0003 | 0.0005 | 0.0002 | 0.0007 | -0.0012 | 0.0024 | 0.0021 | 0.0092 | -0.0017 | 0.0243 |
| 12mo post HCP |  |  |  |  |  |  |  |  |  |  |
| MBS and PBS | -0.0000 | 0.0004 | -0.0002 | 0.0004 | -0.0001 | 0.0005 | 0.0007 | 0.0008 | 0.0003 | 0.0018 |
| l costs | 0.0002 | 0.0006 | 0.0008 | 0.0010 | 0.0007 | 0.0031 | 0.0114 | 0.0087 | -0.0051 | 0.0185 |

*HCP, Home Care Package; MBS, Medicare Benefits Schedule; PBS, Pharmaceutical Benefits Scheme.*
